# Magnetoresponsive fluorescent core–shell nanoclusters for biomedical applications†

**DOI:** 10.1039/d2na00887d

**Published:** 2023-01-31

**Authors:** Giovanni Marco Saladino, Ronak Kakadiya, Shaquib Rahman Ansari, Alexandra Teleki, Muhammet Sadaka Toprak

**Affiliations:** a Department of Applied Physics, Biomedical and X-ray Physics, KTH Royal Institute of Technology SE 10691 Stockholm Sweden saladino@kth.se; b Department of Pharmacy, Science for Life Laboratory, Uppsala University SE 75123 Uppsala Sweden

## Abstract

Nowadays, superparamagnetic iron oxide nanoparticles (SPIONs) have a dominant role in many subfields of biomedicine. Owing to their peculiar properties, they can be employed for magnetic separation, drug delivery, diagnostics, and hyperthermia treatments. However, these magnetic nanoparticles (NPs) suffer from low unit magnetization due to size constraints (up to 20–30 nm) to exhibit superparamagnetic character. In this work, we have designed and synthesized superparamagnetic nanoclusters (SP-NCs) with diameters of up to 400 nm with high unit magnetization for enhanced loading capacity. These were synthesized with conventional or microwave-assisted solvothermal methods, in the presence of either of the two biomolecules (citrate or l-lysine) as the capping agent. Primary particle size, SP-NC size, surface chemistry, and the resultant magnetic properties were observed to be significantly influenced by the choice of synthesis route and capping agent. Selected SP-NCs were then coated with a fluorophore-doped silica shell to provide fluorescence properties, in the near-infrared spectrum region, while silica provided high chemical and colloidal stability. Heating efficiency studies were performed under alternating magnetic field on the synthesized SP-NCs, highlighting their potential in hyperthermia treatment. We envision that their enhanced magnetically-active content, fluorescence, magnetic property, and heating efficiency will pave the way to more effective uses in biomedical applications.

## Abstract 2

Clustered superparamagnetic iron oxide nanoparticles with a fluorophore-doped silica shell for hyperthermia treatment and bioimaging.

## Graphical abstract

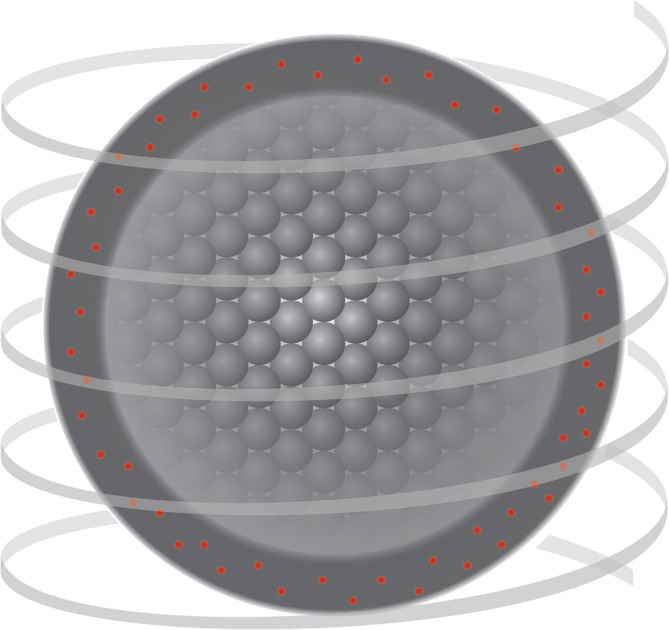

## Introduction

Nowadays, the use of superparamagnetic iron oxide nanoparticles (SPIONs) has spread in many research areas, with their potential application as contrast agents, in controlled drug delivery, molecular imaging, biophotonics, and cancer therapy.^1–7^ Several synthetic routes have been proposed to obtain SPIONs with the desired characteristics to be adapted to their scopes, such as size, morphology, crystal phase, and surface chemistry.^8^ These routes include co-precipitation, solvothermal synthesis, MW-assisted hydrothermal synthesis, thermolysis, and flame spray pyrolysis.^9–12^ One of the limitations of SPIONs is due to the superparamagnetic threshold, which dictates their maximum primary particle size, and consequently, their unit magnetization.^13^ When the nanocrystal size is increased above 20–30 nm, iron oxide nanoparticles (NPs) exhibit ferrimagnetic behavior rather than superparamagnetic.^14^ Thus, other mechanisms are needed to increase the collective size, while still maintaining the superparamagnetic behavior of the primary NPs. The self-assembly of SPION nanoclusters (SP-NCs) is one of the promising routes for their enhanced functionality for biomedical applications.^15–17^ To obtain these SP-NCs, it is usually required to follow multiple-step processes, including embedding in micelles,^18^ polymeric matrices or shells,^19,20^ liposomes,^21^ and hydrogels,^16^ among others. These approaches will unavoidably lead to a reduced unit magnetization due to the presence of a considerable amount of organic content, to stabilize the clusters. Recently, solvothermal synthesis has been revealed as a promising tool for one-step SPION synthesis and clustering, without the need for polymeric or lipidic matrices.^22^ It becomes fundamental to design strategies to prevent NP dissolution and increase biocompatibility. Surface coatings can provide a protective layer and enhance their stability as well as functionality.^23–25^ The constitution of a silica shell has been extensively employed for offering a versatile platform for facile surface modifications. Furthermore, dye-doped core–shell NPs have been demonstrated as dual-mode contrast agents for optical and X-ray fluorescence bioimaging, where silica-coating is responsible for toxicity modulation in biological systems.^23^ In the present work, we design and synthesize self-assembled SP-NCs in a one-pot solvothermal synthesis in ethylene glycol, studying the effect of two different capping agents on the SP-NC size, morphology, and functionality. Furthermore, a modified sol–gel method was used to coat SP-NCs with a dye-doped silica shell, to provide a passivation layer and optical fluorescence properties. With the presented morphological, magnetic, and functional characterization, the obtained core–shell SP-NCs were demonstrated to be a promising theranostic platform combining high magnetically-active content for magnetic hyperthermia treatment and a fluorescent shell for near-infrared bioimaging.

## Experimental

### Materials

Ferric chloride hexahydrate (FeCl_3_·6H_2_O, 97%), sodium acetate anhydrous (NaOAc, >99%), sodium citrate dihydrate (Cit, C_6_H_9_Na_3_O_9_, 99.5%), l-lysine monohydrate (Lys, C_6_H_14_N_2_O_2_, ≥99%), ethylene glycol (EG, >99%), Poly(vinyl-pyrrolidone) (PVP, 55 kDa), Cy5.5 Mono NHS ester (Cy5.5-NHS), (3-aminopropyl)triethoxysilane (APTES, C_9_H_23_NO_3_Si, 99%), triethylamine (TEA, C_6_H_15_N, ≥99%), dimethyl sulfoxide (DMSO, C_2_H_6_OS·H_2_O, ≥99%), hydrochloric acid (HCl, 37%), tetraethyl orthosilicate (TEOS, C_8_H_20_O_4_Si ≥99%), ethanolamine (EA, C_2_H_7_NO, ≥99%) were all purchased from Sigma Aldrich (Stockholm, Sweden). Ethanol absolute (EtOH, ≥99.8%) was obtained from VWR (Stockholm, Sweden). A MilliQ reference water purification system (Merck Millipore) was used for deionized water.

### Nanocluster synthesis

SP-NCs were synthesized *via* a polyol solvothermal method in EG, using FeCl_3_ as the iron precursor, NaOAc as the electrostatic stabilizer, and Lys (or Cit) as the capping agent. A stock solution containing FeCl_3_ (200 mM) and a capping agent (Lys or Cit, 34 mM) in EG (100 mL) was magnetically stirred until all the reagents were fully dissolved. Subsequently, NaOAc (730 mM) was added to the solution, while stirring. For conventional synthesis (ST), part of the solution was transferred to a stainless-steel autoclave with Teflon lining (1/3 of vial volume), and the synthesis reaction was performed at 220 °C for 15 h. Microwave-assisted synthesis (MW) was carried out in the initiator + SP wave (Biotage®, Uppsala, Sweden) microwave reactor, for 2 h at 220 °C (2.45 GHz irradiation), using 1/3 of the vial volume. After cooling down, the as-synthesized SP-NCs were collected and washed with three cycles of EtOH and water, using magnetic separation. Concentrated stocks of lysine-coated (Lys-) and citrate-coated (Cit-) SP-NCs were prepared and stored at 4 °C for further use.

### Fluorophore conjugation

To provide the SP-NCs with optical fluorescence and a passivating/protecting silica coating, Cy5.5-NHS was first conjugated with APTES to act as the dye dopant during the silica condensation reaction.^26^ Briefly, Cy5.5-NHS (1 mg) was dispersed in DMSO (50 μL), together with APTES (0.3 μL) and TEA (0.2 μL). The solution was kept stirring for 24 h in a dark environment and subsequently stored at 4 °C (Cy5.5-APTES).

### Dye-doped silica coating

The SP-NCs synthesized with conventional solvothermal synthesis underwent a coating process with a dye-doped silica shell *via* a modified sol–gel Stöber method, using EA as the base.^23^ Typically, the sol–gel process for silica NP formation includes ammonia as the base.^27^ However, in this work, EA was used for the silica coating process due to its stronger catalytic effect in comparison to ammonia, leading to a faster reaction rate, which can minimize the core dissolution during the coating process.^23^

Lys-SP-NCs underwent a polymer coating process to enhance steric hindrance: from a concentrated stock, Lys-SP-NCs (4.75 mg) and PVP (47.5 mg) were dispersed in water (5 mL), and incubated at room temperature for 1 h, followed by washing *via* magnetic separation.

In a water/EtOH mix (1/3.75), the Lys-SP-NCs were dispersed using a vortex mixer (0.25 mg mL^−1^ in a total of 19 mL). TEOS (50 μL) was introduced, and the dispersion was shaken for 30 min. Cit-SP-NCs were processed the same way but excluding the PVP coating process. Finally, Cy5.5-APTES (1 μL) and EA (200 μL) were added, followed by shaking (2 h). The as-obtained SiO_2_-Cit-SP-NCs and SiO_2_-Lys-SP-NCs were washed by centrifuging with cycles of EtOH (×2) and water (×2), followed by magnetic separation. Finally, the SP-NCs were dispersed in water and stored at 4 °C.

### Characterization techniques

The bare and silica-coated SP-NCs were morphologically and structurally characterized with several techniques. Transmission (JEM-2100F, 200 kV, JEOL) and scanning (FEI Nova 200, 10 kV) electron microscopy (TEM and SEM) were used to evaluate the morphology and dry size of the SP-NCs and primary particle size. SP-NC size (diameter) histograms were fitted with a Gaussian distribution by counting at least 200 SP-NCs from several micrographs with different field-of-views. Drop-casted copper grids and graphite-coated holders were used respectively for TEM and SEM analyses. Dynamic light scattering (DLS) was performed to estimate the hydrodynamic size, polydispersity index (PDI), mean count rate (MCR), and *ζ*-potential (±standard error, in triplicates) to evaluate the SP-NC properties in water and verify the presence of the capping agents, using the Zetasizer Nano ZS90 system (Malvern, UK). Given the high SP-NC uniformity, number-average values and related standard deviation were used and compared to the dry (TEM) size. Photoluminescence spectroscopy (PL, FP-8300, Jasco) was employed to evaluate the optical fluorescence of the silica-coated SP-NCs, owing to the Cy5.5-doping of the silica shell. The crystallographic phase of the SP-NCs was determined using selected-area electron diffraction (SAED), with TEM. Energy-dispersive X-ray spectroscopy (EDX) mapping, with SEM, was used to evaluate the quality of the silica coating. The presence of the capping agents on the NC surface was confirmed with Fourier-transform infrared spectroscopy (FTIR, Thermo Fisher Scientific). Known volumes of stock dispersions were dried *in vacuo* (at 35 °C) to estimate their concentrations (in triplicates). The relative weight of the inorganic content was estimated *via* thermogravimetric analysis (TGA, TGA550, TA Instruments), under nitrogen flow. For the magnetization measurements, a fixed volume (200 μL) with known concentration was dried (35 °C) in 0.2 mL centrifuge tubes and directly inserted in the vibrating sample magnetometer (VSM, EG&G model 155, Princeton Applied Research). To obtain the magnetization curves, the magnetic field was varied up to ±8 kOe. The magnetization was normalized to the inorganic content. For the hyperthermia studies, a dispersion of agarose in water (2%) was heated to 50 °C with magnetic stirring. Once the dispersion turned fully transparent, the corresponding amount of SP-NCs was introduced, to obtain a final NC inorganic concentration of 3 mg mL^−1^, in glass vials, which were sealed after cooling down. These were exposed to an alternating magnetic field (AMF) using magneTherm (Nanotherics Ltd., UK) fitted with a 9-turn coil. For the estimation of the specific absorption rate (SAR) and the intrinsic loss power (ILP), the nominal oscillation frequency was set to 592.2 kHz, and the magnetic field intensity at 14 mT. The heating efficiency was measured as the increase in temperature as a function of time, at room temperature, using a fiber optic probe. After 5 min, the AMF was turned off and the temperature change was recorded for 1 min more. Further information about the estimation of SAR and ILP is presented in ESI† materials.^28^

## Results and discussion

**Fig. 1:**
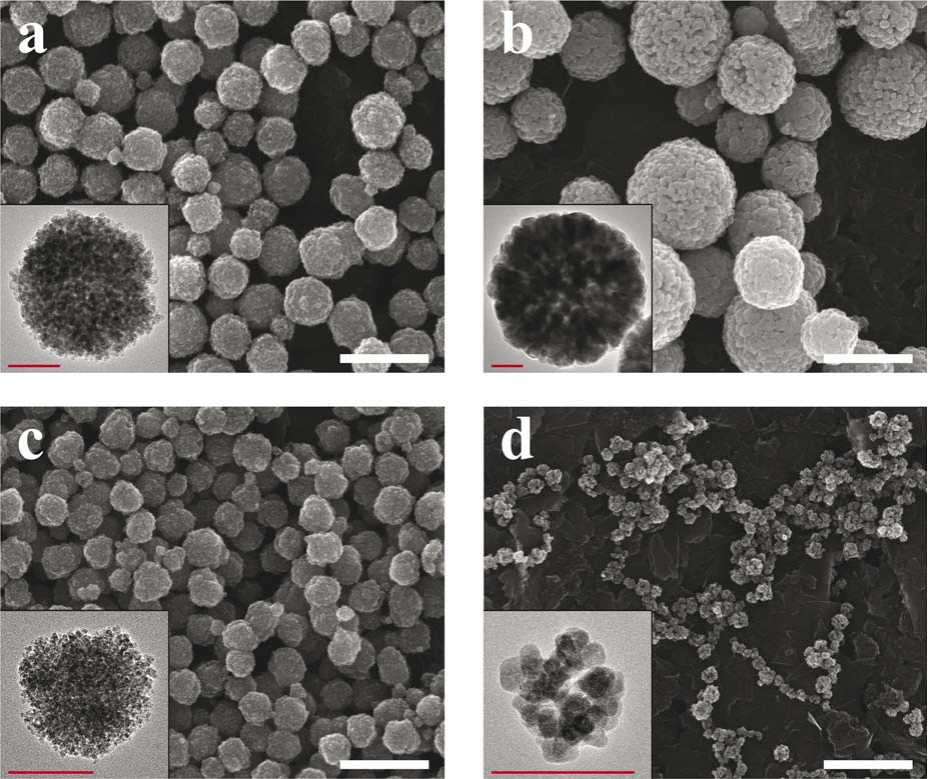
SEM micrographs of the synthesized (a) Cit-SP-NCs (ST), (b) Lys-SP-NCs (ST), (c) Cit-SP-NCs (MW), (d) Lys-SP-NCs (MW). Scale bars indicate 500 nm (in white). In the insets, TEM micrographs of single SP-NCs for the primary particle size estimation. Scale bars in the insets indicate 100 nm (in red).

In the present work, we designed SP-NCs, synthesized with two different capping agents (Cit and Lys), and two synthesis routes: conventional (designated as ST) and MW-assisted solvothermal methods. In Fig. 1, the SP-NC morphology and size are studied with SEM and TEM micrographs. All the synthesized SP-NCs present a well-defined spherical morphology, made up of smaller nanocrystals, except for Lys-SP-NCs (MW), presenting a quasi-spherical morphology. While keeping the same capping agent concentration, temperature, and pressure, Lys-SP-NCs (ST) exhibit the highest NC diameter and primary particle size, as summarized in Table 1.

**Table 1:** Morphological and magnetic properties estimated for the different SP-NC types: NC diameter (*D*_TEM_), primary particle size (*d*_TEM_), hydrodynamic size (*D*_DLS_), saturation magnetization (*M*_S_), coercive field (*H*_C_), and intrinsic loss power (ILP)

	*D*_TEM_ [nm]	*D*_DLS_ [nm]	*d*_TEM_ [nm]	*M*_S_ [emu g^−1^]	*H*_C_ [Oe]	ILP [nH m^2^ kg^−1^]
Cit-SP-NCs (ST)	260 ± 50	270 ± 60	8 ± 2	70	<10	0.10
Cit-SP-NCs (MW)	130 ± 30	200 ± 70	6 ± 2	68	<10	0.18
Lys-SP-NCs (ST)	360 ± 110	400 ± 180	36 ± 10	83	150	0.16
Lys-SP-NCs (MW)	80 ± 20	230 ± 170	14 ± 3	90	50	0.33

Furthermore, the effect of heating homogeneity provided by MW radiation is highlighted when comparing Lys-SP-NCs (MW) with Lys-SP-NCs (ST).

The lower size of Lys-SP-NCs (MW) can be ascribed to a faster nucleation rate and cluster formation, owing to the effective volume heating in MW-assisted synthesis, as well as to the shorter reaction times (2 h for MW and 15 h for ST). When comparing the effect of the different capping agents, the weaker steric and charge hindrance provided by Lys (compared to Cit) favoured the Ostwald ripening and led to larger primary particle sizes, in both MW and ST synthesis. Interestingly, Lys-SP-NCs (MW) exhibited the lowest NC diameter, owing to the lower clustering kinetics in the presence of Lys, compared to Cit. In fact, capping agents are known to play a major role in NP stabilization, particle growth, and biological fate.^29,30^

We speculate that the Lys-SP-NCs (ST) reached the dynamic equilibrium due to the longer reaction times compared to MW synthesis, despite the lower kinetics with Lys. In fact, the SP-NC formation mechanism consists of a two-step growth process, initiated by the SPION nucleation with NaOAc-promoted hydrolysis and supersaturation, and completed by self-assembly at sufficiently high temperatures, mediated by electrostatic interactions and surface tension among nanocrystals.^31^ NaOAc has been demonstrated to regulate the condensation rate by affecting the alkalinity of the reaction system, which directly influences the final SP-NC size.^32^ While SP-NCs synthesized *via* the conventional route were less affected by variations in the reaction temperature (±20 °C), the MW-assisted reaction did not lead to a complete SP-NC formation at temperatures lower than 220 °C (Fig. S1†), indicating the higher influence of the reaction kinetics with reduced reaction times (MW).

**Fig. 2:**
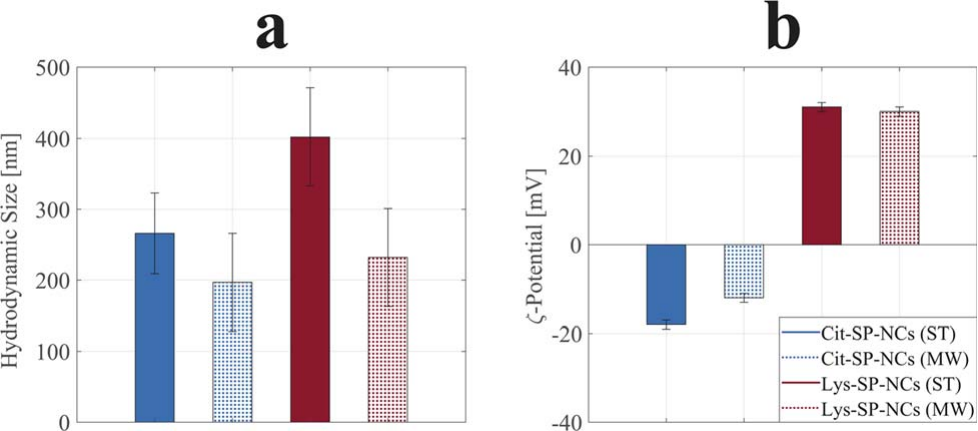
Hydrodynamic sizes (a) and *ζ*-potentials (b) of Cit-SP-NCs (ST) in solid blue, Cit-SP-NCs (MW) in shaded blue, Lys-SP-NCs (ST) in solid red, and Lys-SP-NCs (MW) in shaded red. PDI values were all below 0.2 except for the value of 0.34 obtained for Lys-SP-NCs (MW).

The SP-NCs exhibited a Gaussian size distribution, in contrast to the log-normal trend commonly observed for single nanocrystals synthesized *via* colloidal chemistry.^33^ This observation highlights the presence of two concurrent – but distinct – mechanisms in SP-NC formation: (fast) single-particle nucleation and (slow) clustering reaction in one-pot synthesis.^31^ The hydrodynamic size and *ζ*-potential are important parameters as they significantly influence the SP-NC behaviour in aqueous media, indispensable for potential applications in biological environments. In Fig. 2a, it is possible to observe the hydrodynamic size trend for the four different SP-NCs: in all four cases, it is higher than the dry size estimated by TEM (Fig. S2 and S3†), as it includes the surface functional groups and adsorbed water layers. The PDI was the quantity used to assess the heterogeneity of a sample based on size, yielding values lower than 0.2 for both the SP-NCs (ST). On the contrary, the SP-NCs synthesized using MW-assisted heating present the largest relative deviations from the dry size (Table 1), with the highest detected PDI (0.34) for Lys-SP-NCs (MW), highlighting the presence of some agglomerates in the colloidal form. As smaller SP-NCs have higher surface energies, they possess a higher tendency to reduce the interfacial energy through agglomeration. The MCR analysis featured variations lower than 2% for all the SP-NCs, highlighting their colloidal stability in water. The *ζ*-potential provided the strongest evidence of surface functionalization, confirming the presence of negatively- and positively-charged surfaces in Cit-SP-NCs and Lys-SP-NCs, respectively, for both MW-assisted and conventional synthesis (Fig. 2b). To further confirm the SP-NC surface composition, FTIR spectra of the SP-NCs and related capping agents were collected and analysed (Fig. S4†). Asymmetric and symmetric stretching of the carboxyl groups underwent significant shifts between the unbonded (1580 and 1406 cm^−1^) and bonded (1640 and 1380 cm^−1^) states.^20,34^ The bands between 1100 and 1200 cm^−1^ are ascribed to C–O (Cit and Lys) and C–N (Lys) stretching vibrations. C–H and N–H stretching vibrations can be matched with the weak bands at ≈2900 cm^−1^.^35^ Finally, the Fe–O crystal stretching vibrations are observed at 560 cm^−1^, in all the SP-NC samples.^36^

In Fig. S5,† the electron diffraction (SAED) patterns of the four SP-NC types are presented: they all exhibit the same peculiar pattern, which is associated with magnetite (or maghemite), demonstrating the absence of the other iron oxide crystal phase (hematite), which is not superparamagnetic.^37–39^

The organic content of the SP-NCs was estimated *via* TGA; the resultant data are presented in Fig. S6.† From the analysis, it is fundamental to highlight the increase in organic relative weight with smaller SP-NC diameters; SP-NCs obtained by MW-assisted synthesis exhibited lower inorganic content (81% and 91%) compared to conventional (ST) synthesis (86% and 95%), respectively for Cit- and Lys-SP-NCs. This outcome is ascribed to the decreasing surface-to-volume ratio when increasing the NC size, hence less available surface for surface interactions.

**Fig. 3:**
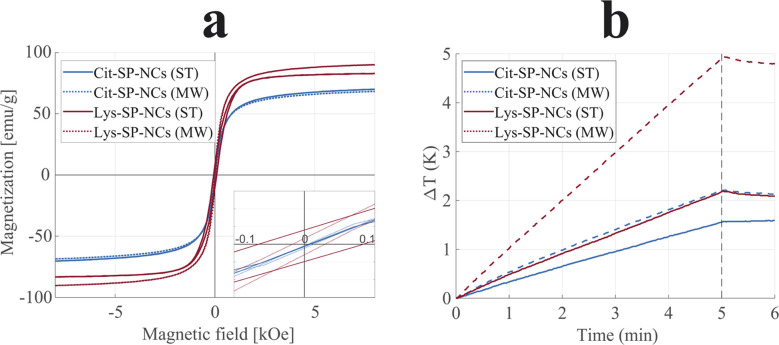
Magnetization curves (a) and heating efficiencies (b) of Cit-SP-NCs (ST), in blue, Cit-SP-NCs (MW), in dashed blue, Lys-SP-NCs (ST), in red, and Lys-SP-NCs (MW), in dashed red. In the VSM inset, a magnified view around the origin highlights the difference in coercivity between the different samples. The SAR values were estimated as 7.5, 13.1, 11.7, and 24.3 W g^−1^ for Cit-SP-NCs (ST), Cit-SP-NCs (MW), Lys-SP-NCs (ST), and Lys-SP-NCs (MW), respectively. At *t* = 5 min, the AMF was turned off.

Comparing the effect of the two capping agents, the combination of larger SP-NCs with a lower molecular weight of Lys (than Cit) led to the highest inorganic – and, thus, the highest magnetically-active – content for Lys-SP-NCs, making them the most promising in terms of unit magnetization, *viz* loading capacity; in ESI materials,† a detailed description of the effect of the mass on the loading capacity is presented. Subsequently, we characterized the synthesized SP-NCs in terms of their magnetic properties. VSM was employed to estimate the saturation magnetization and coercivity of the samples (Fig. 3). Cit-SP-NCs do not exhibit any coercivity (*H*_C_ < 10 Oe), ascribed to the lowest single-particle sizes (*d*_TEM_) equal to 8 and 6 nm, respectively for ST and MW samples. On the contrary, the Lys-SP-NCs present slight coercivity, of 50 Oe (MW) and 150 Oe (ST), directly matching with their increasing single-particle size of 14 and 36 nm, respectively, as summarized in Table 1.

The saturation magnetization (*M*_S_) can be correlated with the primary particle size (*d*_TEM_), where the highest sizes correspond to the highest magnetizations, within a threshold: above that, SPIONs undergo a reduction in saturation magnetization.^40^ Cit-SP-NCs (MW) exhibited the lowest saturation magnetization (68 emu g^−1^), followed by Cit-SP-NCs (ST) with 70 emu g^−1^.

The here presented Lys capping has led to SP-NCs with larger primary particle sizes, hence leading to saturation magnetization values of 90 and 83 emu g^−1^, for Lys-SP-NCs (MW) and Lys-SP-NCs (ST), respectively. These values are equal to 98% and 90% of the bulk magnetization of magnetite, indicating the successful strategy of nanocluster formation to preserve a superparamagnetic behavior while contemporarily increasing the loading capacity, leading to a limited coercivity and absence of permanent attractive interactions between the SP-NCs.

The synthesized SP-NCs were tested for hyperthermia by evaluating their performance in heating efficiency when exposed to an alternating magnetic field, embedded in agar to constitute a tissue-mimicking phantom (Fig. 3b).^41^ All the four tested samples were demonstrated to be suitable for hyperthermia, where the highest value was observed for Lys-SP-NCs (MW), with a SAR value of 22.9 W g^−1^ and ILP of 0.31 nH m^2^ g^−1^ (Table 1). Although the saturation magnetizations of Lys-SP-NCs (ST) and Lys-SP-NCs (MW) are very similar (Fig. 3a), these samples exhibit a significant difference in the heating efficiency (Fig. 3b). This can be attributed to the lower primary particle size of SPIONs in Lys-NC (MW) (∼14 nm), which leads to higher heat generation through relaxation losses. Lys-SP-NCs (ST) are instead affected by hysteresis loss, observed in large primary particle sizes (>30 nm).^42–45^ The heating efficiencies follow a similar trend as the magnetization measurements (VSM), but clearly highlight the superior performances of the samples prepared through MW-assisted synthesis: when comparing its results with the conventional solvothermal synthesis (ST), the obtained SP-NCs exhibit higher saturation magnetizations and heating efficiencies, paving the way to more optimized nanostructures. These results are ascribed both to the optimal primary particle size obtained Lys-SP-NCs (MW) and to the higher crystallinity provided by MW-assisted synthesis.^46^ Nevertheless, the longer reaction times with conventional synthesis permitted to grow bigger SP-NCs, identified as the most promising platforms for bioconjugation in terms of loading capacity. For this reason, Cit-SP-NCs (ST) and Lys-SP-NCs (ST) were selected for the silica coating process to provide them with optical fluorescence properties and a chemically stable platform for further bioconjugation.

**Fig. 4:**
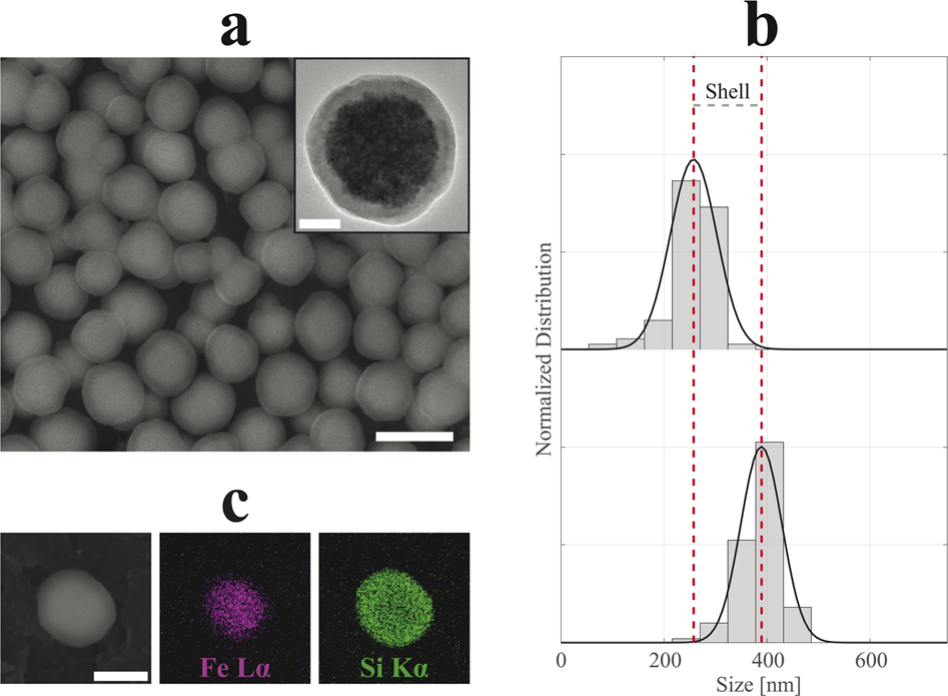
SEM micrograph (a) of SiO_2_-Cit-SP-NCs. Scale bar of 500 nm. In the inset, TEM micrograph of a single-SP-NC with a scale bar of 50 nm. Dry size distribution and Gaussian fit comparison (b) between uncoated (top, *R*^2^ = 95%) and coated (bottom, *R*^2^ = 97%) SP-NCs, highlighting the (double) shell thickness (130 nm). EDX mapping (c) of a single SiO_2_-Cit-SP-NC, detecting Fe Lα and Si Kα characteristic X-ray emission lines. Scale bar indicates 250 nm.

**Fig. 5:**
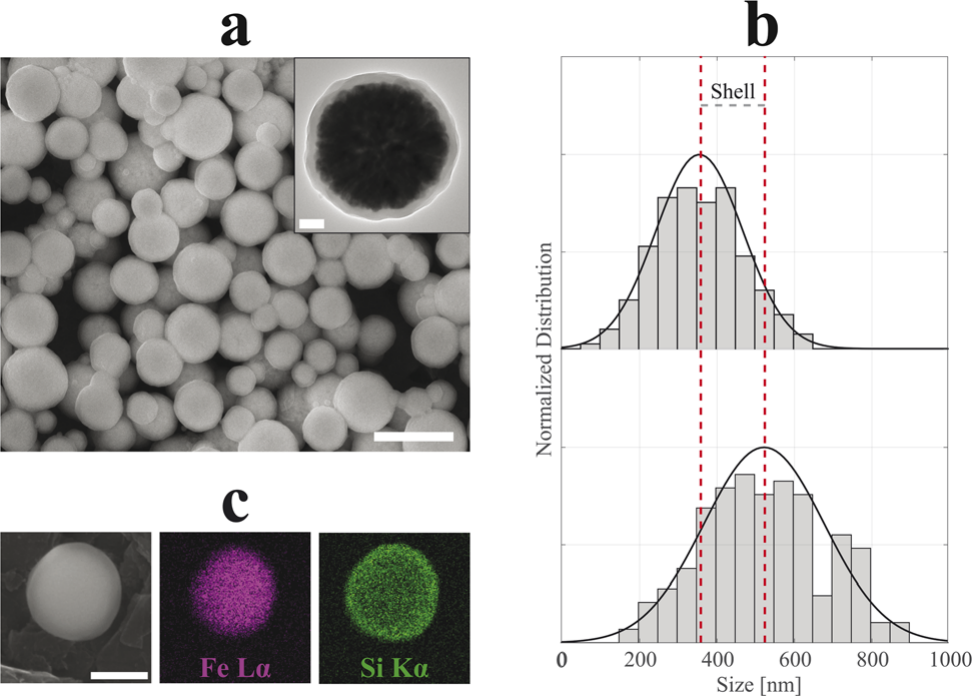
SEM micrograph (a) of SiO_2_-Lys-SP-NCs. Scale bar of 1 μm. In the inset, TEM micrograph of a single-NC with a scale bar of 100 nm. Dry size distribution and Gaussian fit comparison (b) between uncoated (top, *R*^2^ = 98%) and coated (bottom, *R*^2^ = 88%) SP-NCs, highlighting the (double) shell thickness (170 nm). EDX mapping (c) of a single SiO_2_-Lys-SP-NC, detecting Fe Lα and Si Kα characteristic X-ray emission lines. Scale bar indicates 250 nm.

In Fig. 4 and 5, SEM and TEM micrographs of SiO_2_-Cit-SP-NCs and SiO_2_-Lys-SP-NCs are presented, where uniform silica shells of 65 nm and 85 nm coated the Cit-SP-NCs and Lys-SP-NCs, respectively, leading to an overall size of 390 nm and 530 nm while preserving a Gaussian distribution (Table 2). The EDX mapping highlights the main elemental constituents of the silica-coated SP-NCs, through the detection of Fe Lα and Si Kα characteristic X-ray emission lines (Fig. 4c and 5c).

**Table 2:** Morphological and magnetic properties estimated for SiO_2_-Cit-SP-NCs and SiO_2_-Lys-SP-NCs: NC overall diameter (*D*_TEM_), hydrodynamic size (*D*_DLS_), saturation magnetization (*M*_S_), coercive field (*H*_C_), specific absorption rate (SAR), and intrinsic loss power (ILP)

	*D*_TEM_ [nm]	*D*_DLS_ [nm]	*M*_S_ [emu g^−1^]	*H*_C_ [Oe]	SAR [W kg^−1^]	ILP [nH m^2^ kg^−1^]
SiO_2_-Cit-SP-NCs	390 ± 40	410 ± 60	35	<10	4.25	0.06
SiO_2_-Lys-SP-NCs	530 ± 160	690 ± 150	70	80	6.77	0.09

In both cases, the *ζ*-potential measurements highlighted a negatively charged surface, with values of −39 and −29 mV, respectively for SiO_2_-Cit-SP-NCs and SiO_2_-Lys-SP-NCs, where the biggest SP-NCs led to a lower absolute value, due to a lower surface-to-volume ratio. The negative surface charge is a characteristic property of silica NPs, granting high stability in aqueous media.^23,47^ The presence of silica in the dry SP-NC powder is demonstrated with FT-IR spectra for both SiO_2_-Cit-SP-NCs and SiO_2_-Lys-SP-NCs (Fig. S7†), as evidenced by the intense absorption bands due to Si–O–Si stretching vibration localized at 805 and 1085 cm^−1^.^48^ In Fig. S8,† TGA thermograms permitted to estimate the inorganic weight for the two core–shell SP-NCs, pointing up the dehydration and dehydroxylation steps, leading to inorganic relative weights of about 86% for SiO_2_-Cit-SP-NCs and 93% for SiO_2_-Lys-SP-NCs.^49^

**Fig. 6:**
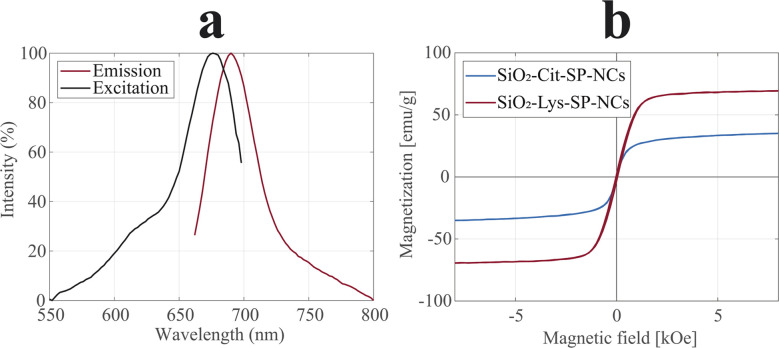
PL spectra (a) of SiO_2_-Lys-SP-NCs, with excitation (black) and emission (red) plots, highlighting excitation maximum at 675 nm and emission maximum at 690 nm. Magnetization curves (b) of SiO_2_-Cit-SP-NCs (blue) and SiO_2_-Lys-SP-NCs (red), normalized to the inorganic content.

The incorporation of the fluorophore (Cy5.5) was attained by cross-linking Cy5.5-NHS with APTES in a pre-step. The introduction of Cy5.5-APTES in the sol–gel reaction did not alter the coating process and the incorporation of the dye in the shell did not lead to fluorescence quenching, in the two coated SP-NCs. The excitation and emission spectra of SiO_2_-Lys-SP-NCs are shown in Fig. 6a, exhibiting maxima at 675 and 690 nm, respectively. These wavelengths, in the near-infrared range, are ideal for *in vitro* and *in vivo* bioimaging applications, since the background autofluorescence impedes the detection of fluorophores in the visible spectrum.^50^

The encapsulation of fluorophore molecules in the silica matrix has been demonstrated to lead to higher values of brightness and photostability, and to enable fluorescence detection at higher depths compared to the free-standing fluorophore.^51–53^

The magnetic properties were analysed in terms of magnetization and heating efficiencies (Fig. 6b and S9†). The coated SP-NCs preserve the peculiar superparamagnetic behaviour, with SiO_2_-Lys-SP-NCs exhibiting a saturation magnetization of 70 emu g^−1^, comparable to the uncoated Cit-SP-NCs.

The SAR and ILP values were affected by a 42% and a 34% reduction compared to the uncoated SP-NCs, for SiO_2_-Cit-SP-NCs and SiO_2_-Lys-SP-NCs, respectively (Tables 1 and 2).

When performing coatings on SPIONs, the magnetic performance is significantly affected due to their high surface-to-volume ratios: single-NP coatings lead to major variations in the overall NP weight. In the present work, the lower surface-to-volume ratio of SP-NCs permits the minimisation of the surface interactions with *e.g.*, coatings and capping agents, thus minimally affecting the magnetic functionality of the final nanostructure.

When analysing the effect of the silica coating on the coercivity, it was observed that SiO_2_-Lys-SP-NCs exhibit a lower coercivity compared to the bare Lys-SP-NCs (ST), 80 and 150 Oe, respectively. This positive outcome was attributed to the quenching of surface-related coercive field contributions, *i.e.*, defect annihilation, as the magnetocrystalline anisotropy is not altered by the silica coating.^54^ The use of agar facilitates the formation of a long-term stable dispersion but leads to the underestimation of the heating efficiencies that these SP-NCs could exhibit in aqueous media. The gelation process inhibits the Brownian motion while preserving the Néel relaxation, due to NC immobilization upon the solidification of agar when cooled down to room temperature.^55^ Nevertheless, the agar phantoms allow the evaluation of the SP-NC heating efficiencies in a tissue-mimicking environment, thus representing a more relevant measurement for biomedical applications.^56^

## Conclusions

In the present paper, we reported the design, synthesis, and characterization of magnetoresponsive core–shell nanoclusters *via* a bottom-up approach. SP-NCs were obtained with both conventional and MW-assisted solvothermal synthesis routes, allowing a critical comparison among the obtained SP-NCs. Their morphological characteristics were assessed to establish the correlation with the exhibited functional magnetic responses. Furthermore, the studies on the hydrodynamic size and surface charge evidenced the high dispersibility of all the obtained SP-NCs. The effect of the capping agent on the SP-NC magnetic and morphological properties was investigated, highlighting the beneficial transition from Cit to Lys for the formation of larger SP-NCs (ST) and bigger primary particle sizes with both the synthesis routes. Moreover, the different functional groups exposed at the SP-NC surface enable a major flexibility for further bioconjugation, *e.g.*, for the scope of magnetic separation, among others. The SP-NC magnetic behaviour and heating efficiency for magnetic hyperthermia were investigated and critically compared, highlighting the dependence on the selected synthesis route and capping agent. The transition from the commonly-employed Cit as the SPION capping agent to Lys led to an improvement of the SP-NC properties in several aspects. Lys-SP-NCs exhibit the best performances in terms of loading capacity, saturation magnetization, and heating efficiency, yielding SP-NCs with an average diameter of 400 nm, achieved for the first time, to the best of the authors' knowledge.

The silica coating was performed with a modified sol–gel method on the pre-synthesized SP-NCs, providing contemporarily a passivation coating and optical fluorescence for envisioned combined bioimaging and therapeutic applications, within the field of theranostics. The here-presented method was demonstrated to be applicable to several SP-NC surfaces, yielding uniform and spherical core–shell SP-NCs. The possibility of SP-NC tracking through their optical fluorescence represents a complementary functionality to hyperthermia treatment. The high penetration depth and induced photostability of the embedded fluorophore (Cy5.5) within the silica shell would permit to study the treatment evolution *in vivo*. All these features pave the way to combined drug delivery, hyperthermia treatment, and near-infrared bioimaging using the developed SP-NC platforms.

## Author contributions

GMS, MST conceived the ideas. GMS, RK designed, synthesized, and characterized the nanomaterials. SA performed the hyperthermia studies. GMS, RK for formal analysis. GMS, AT, MST supervised the work. GMS wrote the original draft. All authors have reviewed and approved the final version of the manuscript.

## Conflicts of interest

The authors declare no competing interests.

## Supplementary Material

NA-005-D2NA00887D-s001
