# Supplementary material for: Magnetoresponsive fluorescent core–shell nanoclusters for biomedical applications
Source: Nanoscale Adv. 2023 Jan 31;5(5):1323–30. doi: 10.1039/d2na00887d (PMC9972542; doi:10.1039/d2na00887d)
Supplement: NA-005-D2NA00887D-s001 [file NA-005-D2NA00887D-s001.pdf]

Electronic Supplementary Material (ESI) for Nanoscale Advances.
This journal is © The Royal Society of Chemistry 2023

Supplementary Materials

Magnetoresponsive Fluorescent Core-shell Nanoclusters for Biomedical Applications

Giovanni Marco Saladino *[a], Ronak Kakadiya [a], Shaquib Ansari [b], Alexandra Teleki [b], and Muhammet Sadaka Toprak *[a]

[1] Department of Applied Physics, Biomedical and X-Ray Physics, KTH Royal Institute of Technology, SE 10691 Stockholm, Sweden

[2] Department of Pharmacy, Science for Life Laboratory, Uppsala University, SE 75123 Uppsala, Sweden.

Supplementary Text

The loading capacity was here defined as the maximum load (m_L) that SP-NCs can tolerate for targeted delivery and drug carriers for biomedical applications, when an external magnetic force is applied (F_M), *viz*:

$$F_M = \nabla(M \cdot B)$$

where $\overline{M}$ and B are the ferrofluid total magnetization ($\overline{M} = m_{NC}M$) and the magnetic flux density, respectively. m_{NC} is the SP-NC magnetic mass. From Newton's Second Law of Motion, in a 1D system, the loaded SP-NCs will undergo an acceleration ($\ddot{x}$), equal to:

$$(m_L + m_{NC})\ddot{x} = m_{NC}\,\nabla(M \cdot B)$$

Assuming to reach the saturation magnetization (M_S) when a magnetic field (H) is applied:

$$(m_L + m_{NC})\ddot{x} = \mu m_{NC} M_S \frac{\partial H}{\partial x}$$

where μ is the magnetic permeability. The acceleration to the system (SP-NC + load) will then be proportional to the NC magnetic mass:

$$\ddot{x} = \frac{m_{NC}}{m_L + m_{NC}} \mu M_S \frac{\partial H}{\partial x}$$

The highest loading capacity will be obtained when maximizing the SP-NC size (and, thus, the magnetically active mass), while minimizing the organic surface and passivation shell content. Besides, the SP-NC unit magnetization can be approximately found as the sum of the constituting NP unit magnetizations ($M_{NC} = \Sigma_i\ M_{NP,i}$), highlighting the importance of the magnetic characteristics of the constituting SPIONs.

The specific absorption rate (SAR) in magnetic hyperthermia[28] was defined as follows:

$$\mathrm{SAR} = \frac{m_{tot}}{m_{NC}} c_p \langle \frac{d(\Delta T)}{dt} \rangle$$

where m_{tot} is the total mass of the tested sample (2 g), c_p is the specific heat capacity of water (4.186 J/g°C), $\Delta T = T - T_0$ is the temperature variation under AMF (T_0 = 22 °C), as a function of time (t), within the first 30 seconds. From the SAR values, the intrinsic loss power (ILP) was estimated:

$$\mathrm{ILP} = \frac{\mathrm{SAR}}{f \cdot H^2}$$

with f = 592.2 kHz (AMF frequency) and H = 14 mT (magnetic field amplitude).

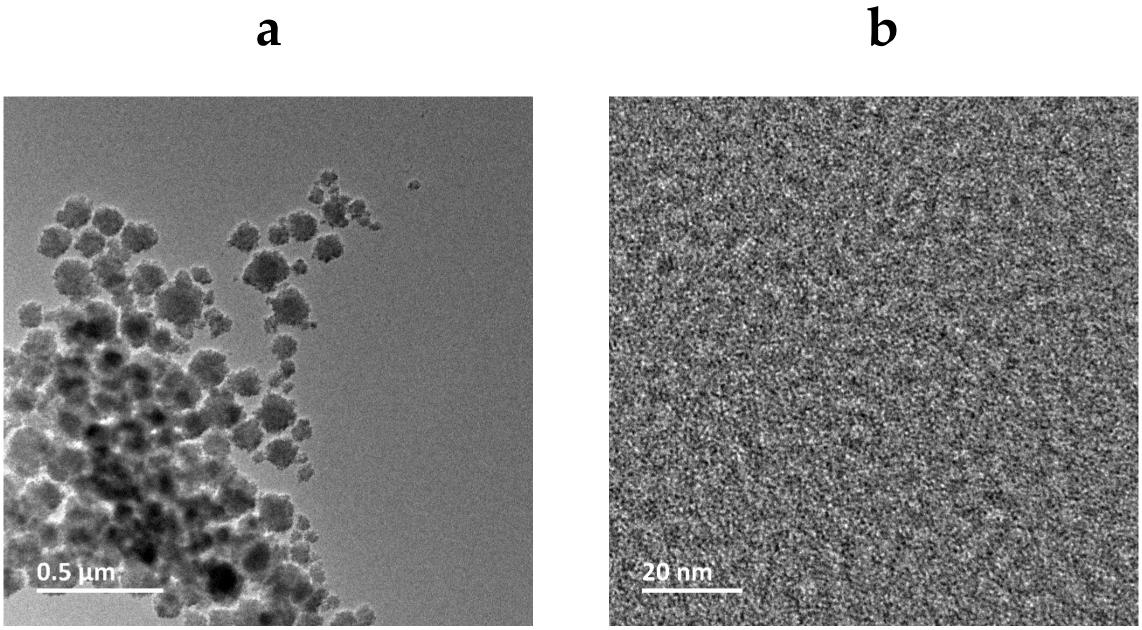

Figure S1. TEM micrographs of uncomplete formation of SP-NCs (MW) in the presence of Cit, using a reaction temperature of 200 °C, highlighting a broad size distribution (**a**) and unclustered SPIONs (**b**).

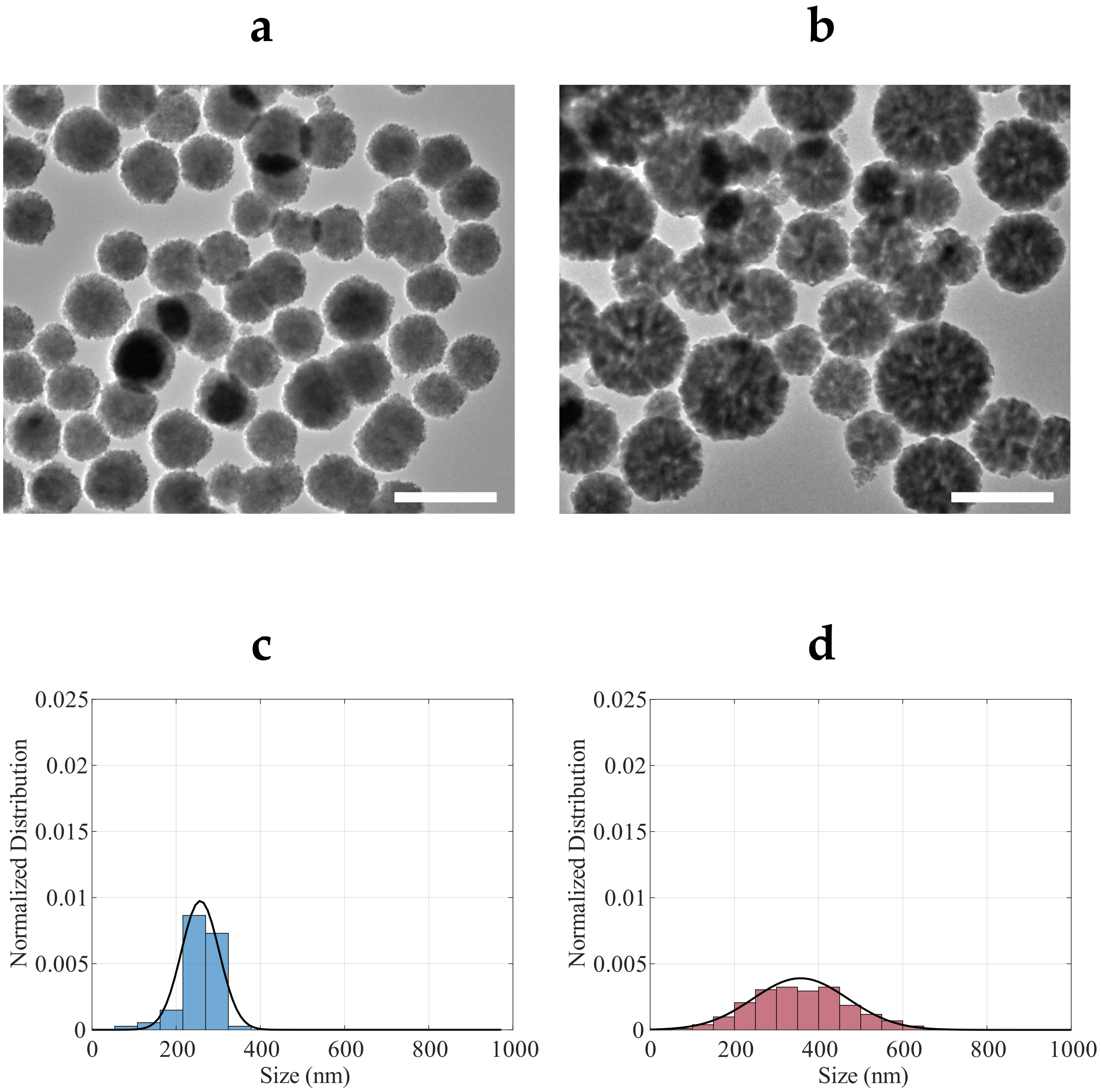

Figure S2. TEM micrographs of Cit-SP-NCs (ST) (**a**) and Lys-SP-NCs (ST) (**b**). Scale bar of 500 nm. Dry size distribution and gaussian fit comparison of Cit-SP-NCs (ST) (**c**) with R^2 = 95% and of Lys-SP-NCs (ST) (**d**) with R^2 = 98%.

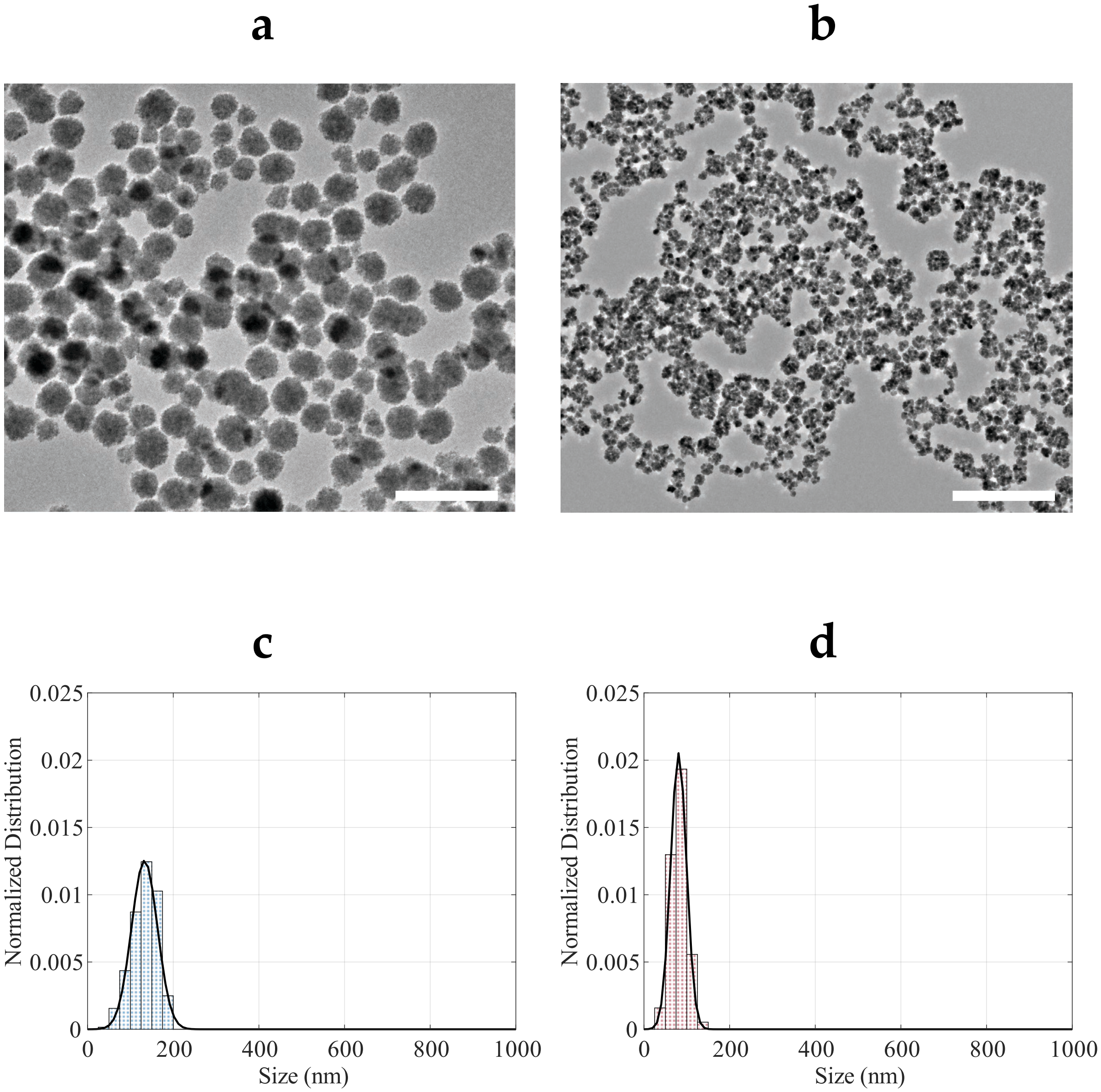

Figure S3. TEM micrographs of Cit-SP-NCs (MW) (**a**) and Lys-SP-NCs (MW) (**b**). Scale bar of 500 nm. Dry size distribution and gaussian fit comparison of Cit-SP-NCs (MW) (**c**) with R^2 = 97.4% and of Lys-SP-NCs (MW) (**d**) with R^2 = 99.7%.

a

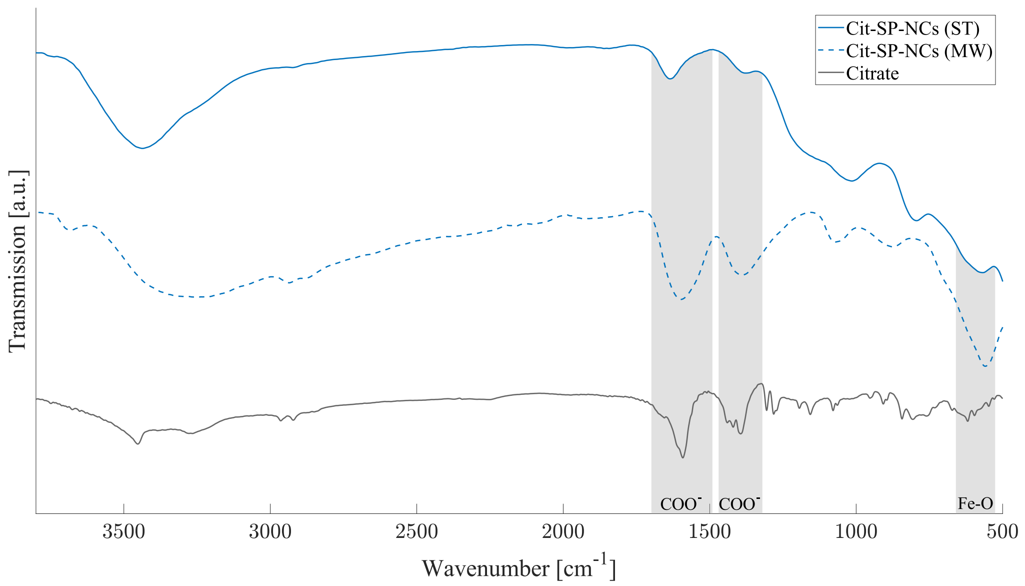

b

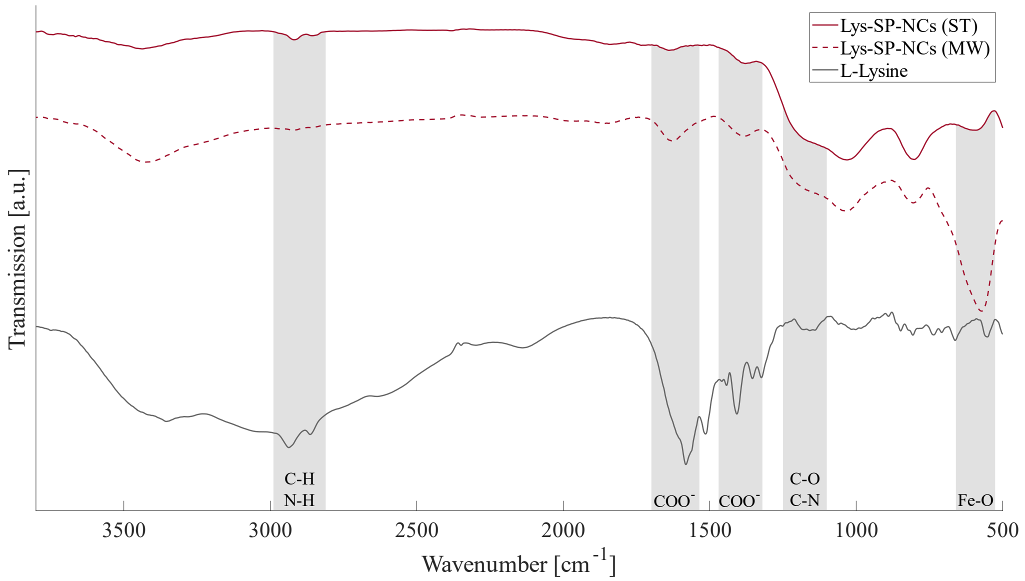

Figure S4. FT-IR spectra for the synthesized NCs and corresponding capping agent: Cit-SP-NCs (ST), Cit-SP-NCs (MW), and Citrate (**a**); Lys-SP-NCs (ST), Lys-SP-NCs (MW), and L-Lysine (**b**).

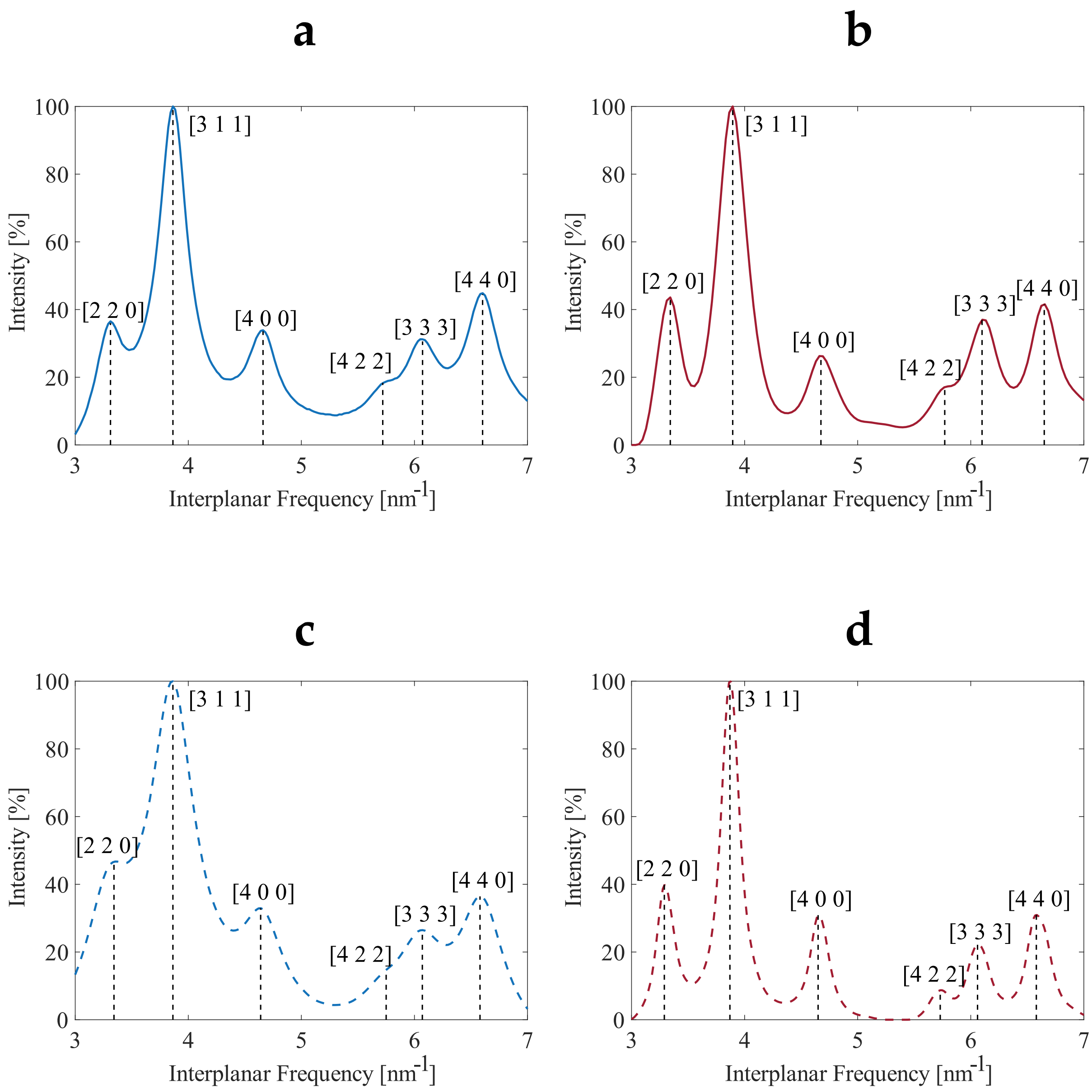

Figure S5. Linear diffraction profiles obtained from the selected area electron diffraction (SAED) of Cit-SP-NCs (ST) (**a**), Lys-SP-NCs (ST) (**b**), Cit-SP-NCs (MW) (**c**), and Lys-SP-NCs (MW) (**d**). The peaks reveal the fcc structure of the constituting SPIONs (magnetite/maghemite, CODs 1011032/9006316) in all the four synthesized SP-NCs.

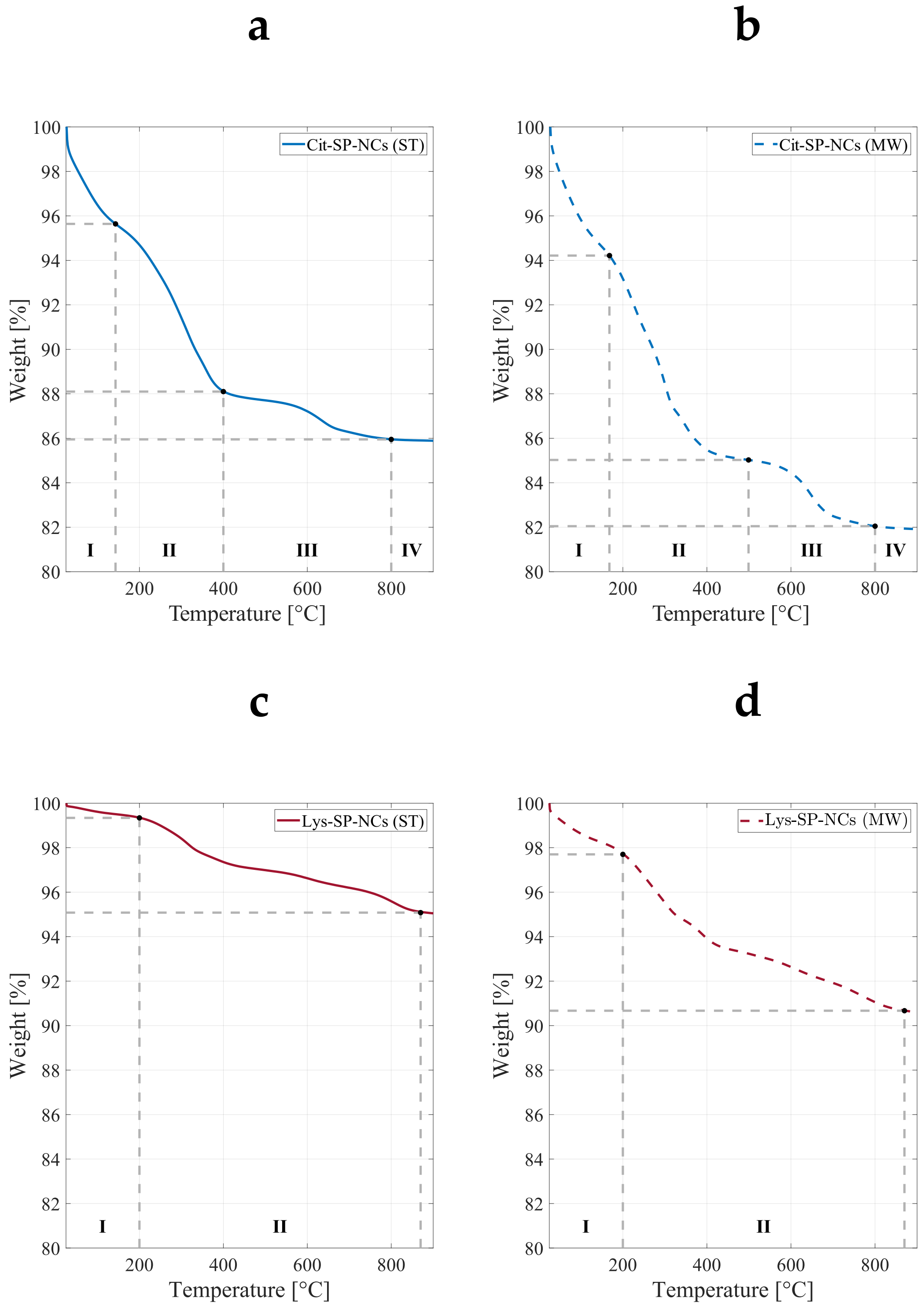

Figure S6. TGA thermograms of Cit-SP-NCs (ST) (**a**), Lys-SP-NCs (ST) (**b**), Cit-SP-NCs (MW) (**c**), and Lys-SP-NCs (MW) (**d**), revealing the weight percentage of the organic and inorganic content. Below 200 °C, water desorption is detected (step I). The weight loss between 200 and 850 °C is ascribed to the pyrolysis of citrate (**a**, **b**) and L-Lysine (**c**, **d**) on the SP-NC surface.

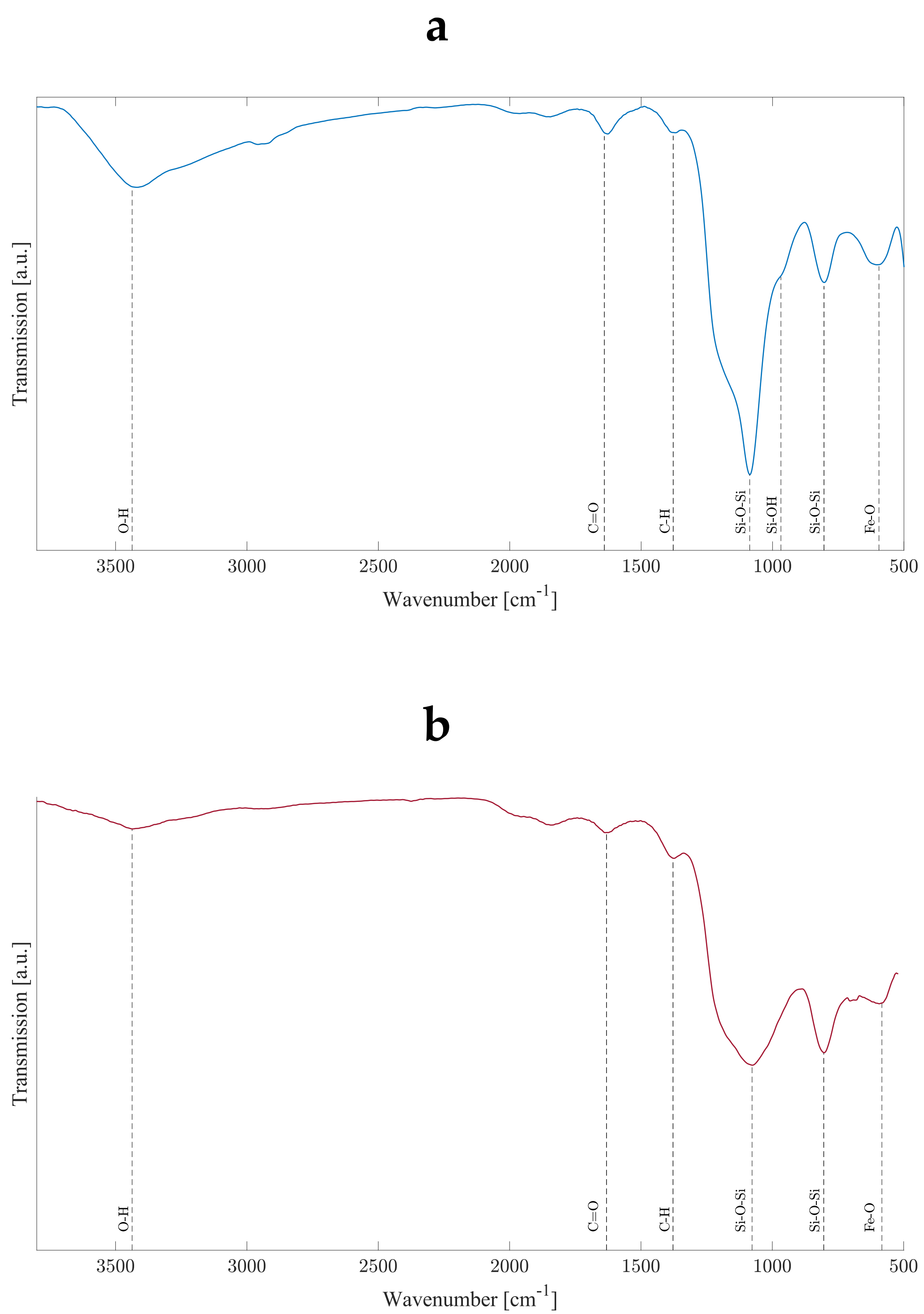

Figure S7. FT-IR spectra for the SiO_2-Cit-SP-NCs (**a**) and SiO_2-Lys-SP-NCs (**b**).

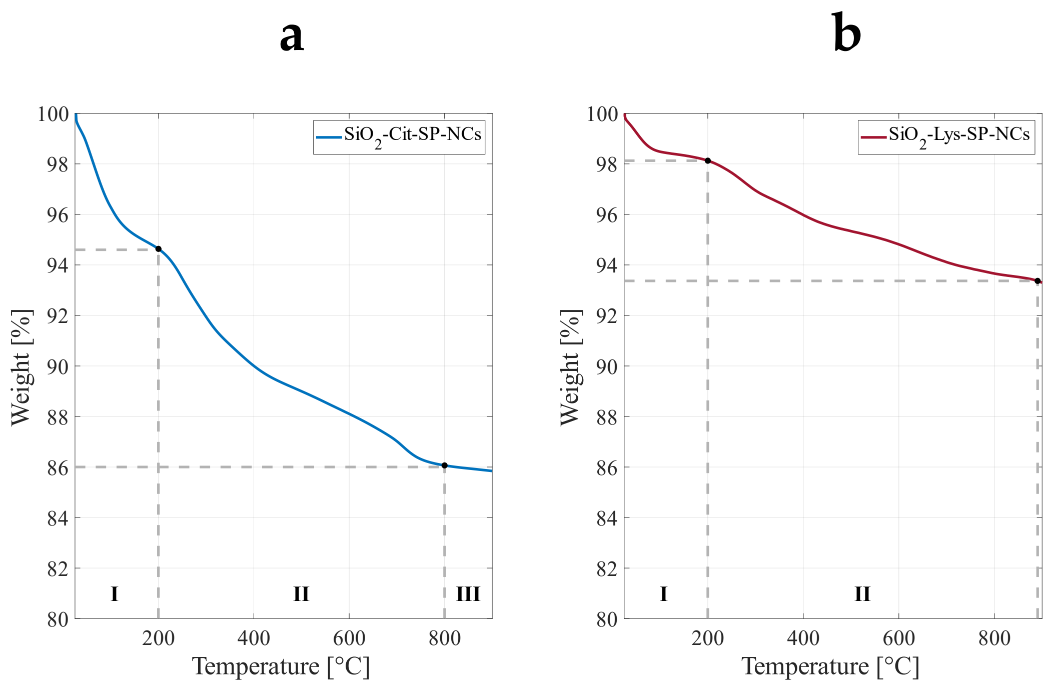

Figure S8. TGA thermograms of SiO_2-Cit-SP-NCs (**a**) and SiO_2-Lys-SP-NCs (**b**), revealing the weight percentage of the organic and inorganic content. Below 200 °C, water desorption is detected (step I). The weight loss between 200 and 850 °C is ascribed to the dehydroxylation process on the NC surface.

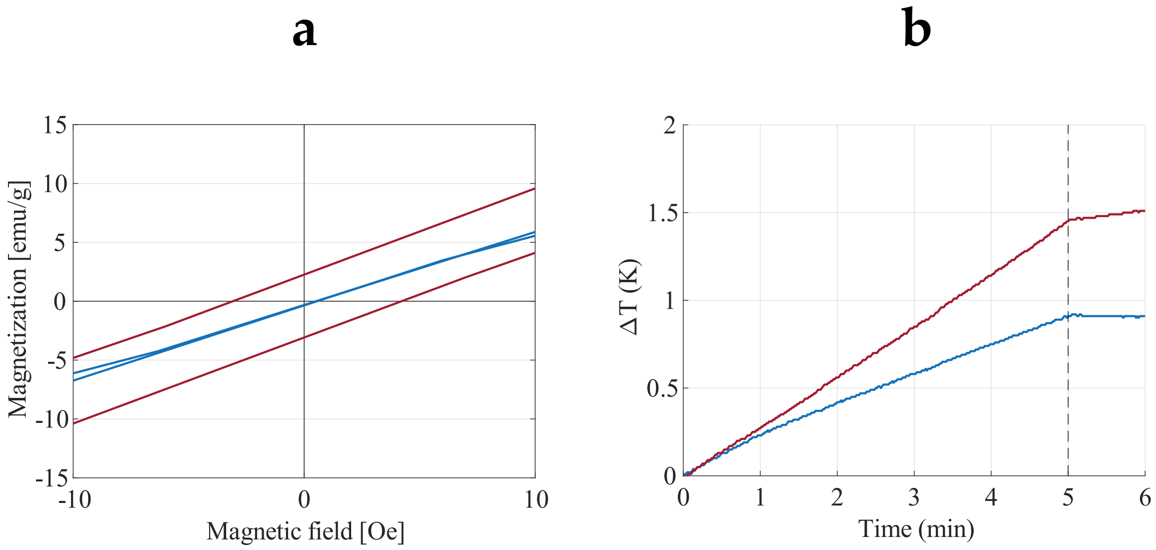

Figure S9. Magnified magnetization curve (**a**) centered in the origin, highlighting the difference in coercivity between SiO_2-Cit-SP-NCs (blue) and SiO_2-Lys-SP-NCs (red). Heating efficiencies (**b**) of SiO_2-Cit-SP-NCs (blue) and SiO_2-Lys-SP-NCs (red). The SAR values were estimated as 4.25 and 6.77 W/g for SiO_2-Cit-SP-NCs and SiO_2-Lys-SP-NCs, respectively, considering the overall inorganic weight. At t = 5 min, the AMF was turned off.
